# Body roundness index and cognitive function in older adults: a nationwide perspective

**DOI:** 10.3389/fnagi.2024.1466464

**Published:** 2024-11-20

**Authors:** Feng Zhang, Zhongxing Ning, Can Wang

**Affiliations:** ^1^Department of Neurology, The Affiliated Nanhua Hospital, Hengyang Medical School, University of South China, Hengyang, Hunan, China; ^2^Department of Intensive Care Unit, Guangxi Hospital Division of The First Affiliated Hospital, Sun Yat-sen University, Nanning, China

**Keywords:** NHANES, cognitive function, obesity, older, body roundness index

## Abstract

**Background:**

Obesity negatively impacts cognitive function. However, the correlation between the body roundness index (BRI) and cognitive performance remains inadequately explored.

**Methods:**

This study used data from the National Health and Nutrition Examination Survey (NHANES) collected from 2011 to 2014 to examine the correlation between BRI and cognitive function in individuals aged 65 and older. Models of multiple linear regression were used to investigate the relationship between BRI and cognitive performance. Additionally, smoothed curve fittings explored potential non-linear associations. Interaction tests and subgroup analyses were also performed.

**Results:**

One thousand eight hundred seventy participants were taken into account, revealing an important negative relationship between BRI levels and cognitive performance. In the fully adjusted model, elevated BRI was substantially correlated with lower Digit Symbol Substitution Test (DSST) scores (*β* = −0.34, 95% CI = −0.64 to −0.05, *p* = 0.023), indicating that the higher BRI values are linked to worse cognitive performance. Sex differences were observed, with males showing a stronger negative association (*p* for interaction = 0.040).

**Conclusion:**

Elevated BRI is related to worse cognitive function in the elderly population.

## 1 Introduction

With the global aging population, the prevalence of dementia is expected to increase significantly (Ahmadi-Abhari et al., 2017), affecting an estimated 152 million individuals by 2050 (Nichols et al., 2022). Dementia, characterized by progressive cognitive decline and memory loss, severely impairs daily activities. Alzheimer’s disease (AD), in particular, has become a major global public health challenge (Wimo et al., 2023; Feigin et al., 2021). Despite extensive research over the past few decades, no effective treatment for dementia has been discovered. Cognitive decline is recognized as a key predictor of dementia. Thus, it is essential to discover modifiable risk factors linked to cognitive impairment in order to reduce the occurrence of dementia.

Obesity, a growing global issue, can disrupt brain homeostasis and adversely affect the central nervous system and cognitive function (O’Brien et al., 2017). Studies have demonstrated a significant association between obesity and cognitive impairment (Beeri et al., 2022). Furthermore, obesity is closely associated with AD, a specific subtype of dementia (Livingston et al., 2020; Anstey et al., 2011; Arnold et al., 2018). However, traditional obesity indicators such as waist circumference (WC) and body mass index (BMI) have limitations in accurately reflecting fat distribution (Li et al., 2024). To address these limitations, Thomas et al. developed the body roundness index (BRI), a more precise method for evaluating body and visceral fat (Thomas et al., 2013). Compared to BMI, BRI incorporates both weight and WC, enabling a more accurate evaluation of body fat and visceral fat distribution (Rico-Martín et al., 2020; Tang et al., 2021). Studies indicate that visceral fat is metabolically active and is closely associated with metabolic disorders like insulin resistance, dyslipidemia, and inflammation, all of which significantly increase the risk of cognitive decline (Zeng et al., 2023). Therefore, as a marker of visceral fat, BRI may outperform traditional obesity measures (such as BMI) in predicting cognitive decline. However, the research linking BRI to cognitive function is still limited, necessitating further studies to confirm its validity.

This research analyzes data from the National Health and Nutrition Examination Survey (NHANES) to examine the correlation between BRI and cognitive performance in older persons in the United States. The objective is to get a better understanding of early diagnosis of dementia.

## 2 Materials and methods

### 2.1 Survey description

The NHANES survey, conducted by the National Center for Health Statistics (NCHS), is an extensive nationwide study that evaluates the health and nutritional status of Americans (Johnson et al., 2014). This survey operates on a two-year cycle, using representative samples and a complicated multi-stage stratified random sampling approach. Participants had a home interview initially, and subsequently visited a mobile examination center (MEC) for a health assessment. The NCHS Research Ethics Review Board granted authorization for the research protocol, and each participant provided signed informed consent. Detailed information about the data is available on the NHANES official website^1^. The STROBE standards for cross-sectional research were followed in this investigation.

### 2.2 Study population

**Figure 1:**
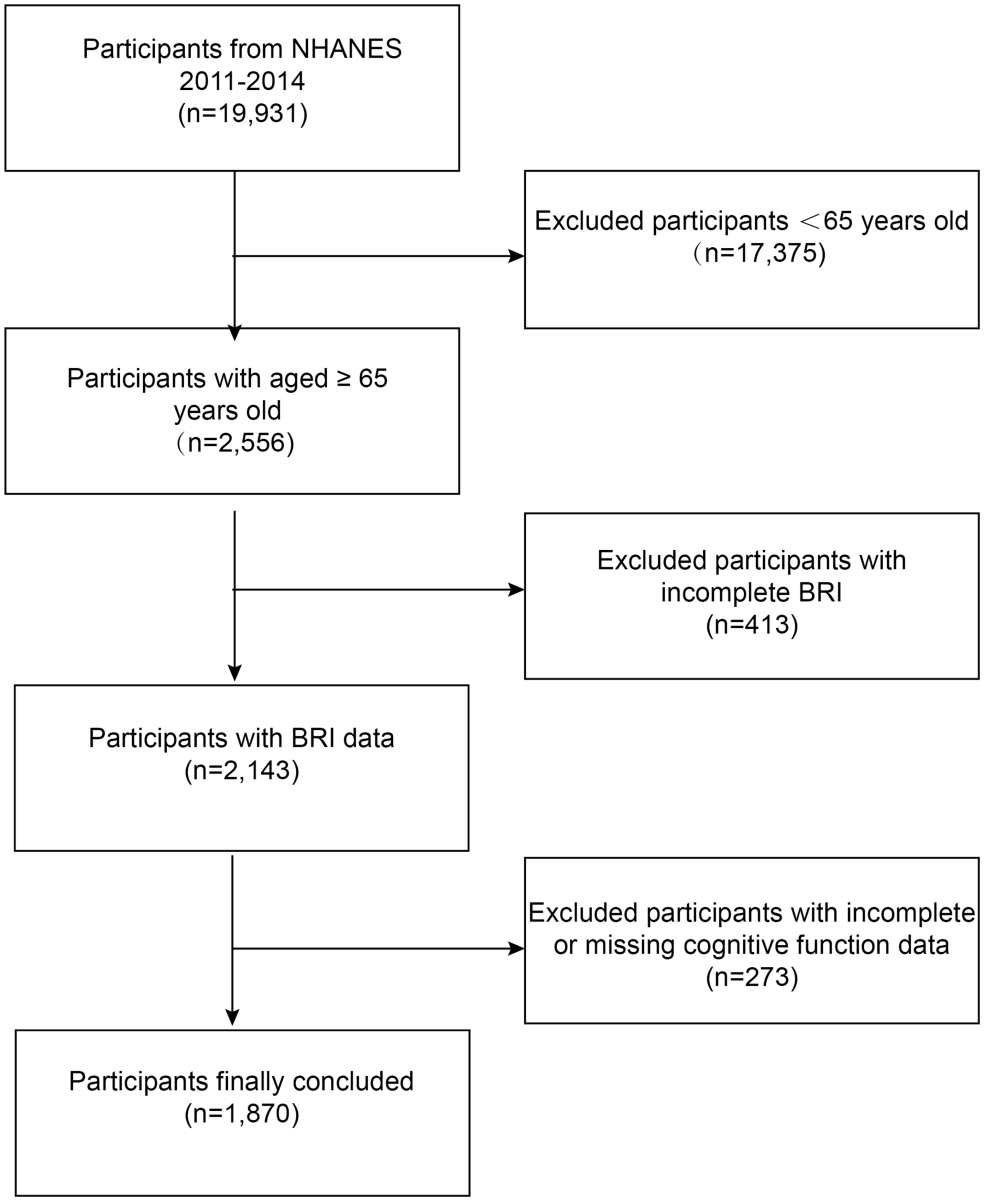
Flowchart of participant selection. NHANES, National Health and Nutrition Examination Survey; BRI, body roundness index.

Data from the NHANES cycles 2011–2012 and 2013–2014 were used in this investigation. From 2011 and 2014, 19,931 participants enrolled in the NHANES. Adults 65 years of age and older who completed the MEC cognitive functioning survey were the subjects of this research, with an initial sample size of 2,556. Individuals with incomplete BRI data (*n* = 413) and those with incomplete or inaccurate cognitive test scores (*n* = 273) were excluded. After these exclusions, the final size was 1,870 individuals (Figure 1).

### 2.3 Assessment of body roundness index

The BRI is a novel metric for assessing body shape, calculated using height (cm) and WC (cm). Data on height and WC were collected from the examination records of the subjects. Measurements were taken by certified health specialists with assistance from assistants at mobile testing facilities to guarantee accuracy. Participants removed their clothing and shoes before measurements. Height was measured as standing height, while WC was evaluated at the level midway between the lower rib and the top of the hip while standing. The formula for calculating BRI is as follows (Thomas et al., 2013):


$$\begin{array}{l} BRI=364.2–365.5\times \\ \surd \left(1-{\left[WC\;\text{in centimeters}/2\pi \right]}^{2}/{\left[0.5\times \text{height in meters}\right]}^{2}\right)\text{.} \end{array}$$

The higher BRI values indicate greater obesity. Due to the lack of clear reference ranges, this study divided BRI into quartiles based on previous literature to analyze its association with cognitive function (Zhou et al., 2022).

### 2.4 Assessment of cognitive function

This study used three assessments to evaluate cognitive function: the Consortium to Establish a Registry for Alzheimer’s Disease Word List (CERAD W-L), the Animal Fluency Test (AFT), and the Digit Symbol Substitution Test (DSST) (Morris et al., 1989).

The CERAD W-L uses one delayed recall test and three quick recall trials to evaluate a person’s ability to learn and remember new verbal knowledge (Fillenbaum and Mohs, 2023; Paajanen et al., 2014). This exam has a total score range of 0–40. The AFT is designed to assess categorical verbal fluency (Ardila, 2020). Participants had 1 min to list as many animals as they could, with each correctly named animal earning one point. Scores for the AFT range from 3 to 39. The DSST, adapted from the Wechsler Adult Intelligence Scale, is a cognitive performance test that evaluates processing speed, sustained attention, and working memory. It functions as an experimental instrument for comprehending associative learning in humans (Jaeger, 2018). Participants completed a two-minute task of replicating graphic symbols in 133 boxes. Scores for the DSST range from 0 to 105.

In accordance with prior studies, the cut-off values for low cognitive functioning were set at 21 for CERAD W-L, 13 for AFT, and 34 for DSST (Chen et al., 2017). Lower scores on these tests indicate poorer cognitive functioning (Wang et al., 2024).

### 2.5 Covariates

After reviewing the available literature, many possible factors that may influence the outcome were examined (Jia et al., 2024). These factors included age, sex (male or female), race (Mexican American, other Races, non-Hispanic White individuals, or non-Hispanic Black individuals), education level (below high school, high school, or above high school), poverty income ratio (PIR). Smoking status was assessed by asking participants, “Do you currently smoke?” Those who responded with ‘1’ (every day) or ‘2’ (some days) were categorized as ‘current smokers,’ while all other responses were classified as ‘non-smokers.’ Alcohol consumption was assessed by asking, “How often did you drink alcohol in the past 12 months?” Those who gave a numeric response were categorized as ‘drinkers,’ while others were classified as ‘non-drinkers.’ Hypertension, hyperlipidemia, diabetes, and stroke were obtained through self-reports from the subjects, specifically inquiring whether they had ever been diagnosed with these conditions by a physician. Depression symptoms were assessed with the Patient Health Questionnaire-9 (PHQ-9), which evaluates the frequency of symptoms over the past 2 weeks. Response options ranged from 0 to 3, with a total score of PHQ-9 ≥ 10, indicating depression, a threshold with high sensitivity and specificity (Kroenke et al., 2001). All data were obtained through standardized questionnaires, physical examinations, and laboratory examinations collected by licensed healthcare professionals. The questionnaires were administered via face-to-face interviews to ensure participants provided accurate responses relevant to the study. Detailed measuring protocols for these variables can be found on the official NHANES website.

### 2.6 Statistical analysis

R (The R Foundation, Vienna, Austria) and Empower (X&Y Solutions, Boston, MA, United States) were used for statistical analyses. Continuous variables were shown in terms of means with standard deviations (SD) in the baseline characteristics table; categorical variables were presented as percentages (%). The random forest imputation method, implemented with the missForest package (version 1.4) in R, was used to impute missing covariates. This approach enhances statistical efficiency and reduces bias by utilizing the relationships among the available variables. Given the complex sampling design of NHANES, all statistical analyses followed CDC analytical guidelines, incorporating sample weights, clustering, and stratification. NHANES survey weights adjust for unequal selection probabilities, non-response, and post-stratification to ensure the results represent the U.S. civilian non-institutionalized population. The relationship between BRI and cognitive function was analyzed using three distinct weighted multivariate linear regression models. Model 1 did not involve adjustments for covariates. Model 2 adjusted for age, sex, and race. Model 3 adjusted for age, sex, race, education level, PIR, alcohol and smoking use status, hypertension, hyperlipidemia, diabetes, stroke, and depression. After converting the BRI score from a continuous variable to quartiles, trend tests were conducted to assess linear correlations between BRI and cognitive function. Weighted subgroup analyses of the association between BRI and cognitive function were conducted using stratification factors including sex, race, education level, alcohol and smoking use status, hypertension, hyperlipidemia, diabetes, stroke, and depression. Interaction tests were applied to evaluate the consistency of this association across different groups. In addition, the weighted smooth curve fitting was used to examine the non-linear relationship between BRI and cognitive performance. A two-tailed *p* value below 0.05 was regarded as statistically significant.

## 3 Results

### 3.1 Characteristics of the study population

One thousand, eight hundred seventy individuals aged 65 years and older were enrolled in this research; their weighted mean age was 72.72 years (SD = 5.32), and 51.18% of them were female (weighted percentage). Participants were stratified into four quartiles according to their BRI values: 1.44–4.53 (Quartile 1), 4.53–5.69 (Quartile 2), 5.69–7.03 (Quartile 3), and 7.03–15.42 (Quartile 4). Weighted analyses showed that individuals in the highest quartile of the BRI were more likely to be female, have higher educational levels, consume alcohol more frequently, and smoke less. They also had higher rates of hypertension and hyperlipidemia, but lower rates of diabetes, stroke, and depression. Additionally, they were more likely to belong to lower-income households compared to those in the lowest quartile. Moreover, these individuals scored lower on the DSST (Table 1).

**Table 1:** Basic characteristics of participants by BRI tertiles among U.S. older adults.

	Body roundness index				
Variables	Q1 (1.44–4.53) (*n* = 468)	Q2 (4.53–5.69) (*n* = 467)	Q3 (5.69–7.03) (*n* = 467)	Q4 (7.03–15.42) (*n* = 468)	*p*
Age, years	72.49 ± 5.54	72.88 ± 5.34	72.42 ± 5.12	72.01 ± 5.16	0.098
Sex, %					<0.001
Male	41.80	51.83	47.32	39.39	
Female	58.20	48.17	52.68	60.61	
Race, %					0.295
Mexican American	1.48	2.65	3.27	4.34	
Other Races	9.85	9.76	7.48	8.33	
Non-Hispanic White	80.12	81.00	82.35	79.40	
Non-Hispanic Black	8.55	6.59	6.90	7.93	
Education, %					<0.001
<High school	11.86	19.26	17.85	21.73	
High school	19.08	22.16	24.95	22.68	
>High school	69.07	58.58	57.20	55.60	
Alcohol, %					0.002
No	36.05	34.36	42.29	44.92	
Yes	63.95	65.64	57.71	55.08	
Smoking, %					0.025
No	88.08	90.60	92.95	92.91	
Yes	11.92	9.40	7.05	7.09	
Hypertension, %					<0.001
No	53.19	44.82	32.58	25.85	
Yes	46.81	55.18	67.42	74.15	
Hyperlipidemia, %					<0.001
No	49.84	37.02	34.47	37.29	
Yes	50.16	62.98	65.53	62.71	
Diabetes, %					<0.001
No	91.48	85.78	74.98	68.12	
Yes	8.52	14.22	25.02	31.88	
Stroke, %					0.018
No	91.64	94.68	94.62	90.29	
Yes	8.36	5.32	5.38	9.71	
Depression, %					<0.001
No	94.85	95.20	94.23	88.20	
Yes	5.15	4.80	5.77	11.80	
PIR	3.19 ± 1.46	3.16 ± 1.52	2.90 ± 1.46	2.64 ± 1.50	<0.001
CERAD W-L	25.57 ± 7.00	24.76 ± 6.11	25.37 ± 6.21	25.30 ± 5.96	0.238
AFT	17.72 ± 5.52	17.34 ± 5.62	17.68 ± 5.01	17.39 ± 5.76	0.608
DSST	51.33 ± 16.38	50.05 ± 15.11	49.26 ± 15.65	47.03 ± 15.71	<0.001

Mean ± SD for continuous variables; the *p* was calculated by the weighted linear regression model; (%) for categorical variables; the *p-*value was calculated by the weighted chi-square test. Q, Quartile; PIR, poverty income ratio; CERAD W-L, Consortium to Establish a Registry for Alzheimer’s Disease Word List; AFT, Animal Fluency Test; DSST, Digit Symbol Substitution Test.

### 3.2 Association between BRI and cognitive function

Weighted regression analysis (Table 2) demonstrated a negative correlation between BRI levels and DSST scores. This relationship remained significant after adjusting for all confounding factors (*β* = −0.34, 95% CI = −0.64 to −0.05, *p* = 0.023). Treating BRI as a categorical variable (quartiles) confirmed the persistence of this significant association in both unadjusted models and in models adjusted for key demographic factors (*p* for trend <0.001). However, the increase was not significant (*p* for trend = 0.302) after correcting for all covariates. There were no significant relationships between BRI and the CERAD W-L or AFT scores. These weighted results imply that a greater BRI is linked to a lower level of cognitive function, particularly as measured by the DSST, indicating that a higher BRI may negatively impact cognitive performance.

**Table 2:** Associations between the BRI with cognitive function.

Cognitive function	Model1 [*β*(95%CI)]	*p*	Model2 [*β*(95%CI)]	*p*	Model3 [*β*(95%CI)]	*p*
CERAD W-L	−0.07 (−0.22, 0.07)	0.322	−0.15 (−0.29, −0.01)	0.033	−0.04 (−0.19, 0.10)	0.541
Q1	Ref		Ref		Ref	
Q2	−0.82 (−1.63, −0.00)	0.049	−0.45 (−1.20, 0.31)	0.248	−0.17 (−0.92, 0.57)	0.655
Q3	−0.21 (−1.01, 0.60)	0.617	−0.14 (−0.89, 0.61)	0.720	0.28 (−0.48, 1.03)	0.468
Q4	−0.28 (−1.09, 0.54)	0.509	−0.47 (−1.23, 0.29)	0.230	0.17 (−0.61, 0.95)	0.671
*p* for trend		0.863		0.375		0.456
AFT	−0.04 (−0.16, 0.09)	0.570	−0.07 (−0.18, 0.05)	0.274	0.10 (−0.02, 0.22)	0.101
Q1	Ref		Ref		Ref	
Q2	−0.39 (−1.09, 0.32)	0.283	−0.39 (−1.04, 0.25)	0.235	−0.07 (−0.70, 0.55)	0.824
Q3	−0.04 (−0.73, 0.66)	0.919	−0.20 (−0.84, 0.44)	0.534	0.39 (−0.24, 1.02)	0.228
Q4	−0.33 (−1.04, 0.38)	0.358	−0.47 (−1.12, 0.18)	0.161	0.41 (−0.24, 1.06)	0.219
*p* for trend		0.575		0.249		0.114
DSST	−0.92 (−1.28, −0.56)	<0.001	−1.05 (−1.37, −0.73)	<0.001	−0.34 (−0.64, −0.05)	0.023
Q1	Ref		Ref		Ref	
Q2	−1.28 (−3.30, 0.73)	0.213	−0.63 (−2.39, 1.13)	0.482	0.52 (−1.03, 2.07)	0.510
Q3	−2.07 (−4.07, −0.06)	0.043	−2.16 (−3.91, −0.42)	0.015	0.22 (−1.34, 1.79)	0.780
Q4	−4.29 (−6.32, −2.27)	<0.001	−4.66 (−6.43, −2.89)	<0.001	−0.83 (−2.45, 0.79)	0.317
*p* for trend		<0.001		<0.001		0.302

Model 1: no covariates were adjusted. Model 2: age, sex, and race were adjusted. Model 3: age, sex, race, education level, PIR, alcohol and smoking use status, hypertension, and hyperlipidemia, diabetes, stroke, and depression were adjusted. Q, Quartile; PIR, poverty income ratio; CERAD W-L, Consortium to Establish a Registry for Alzheimer’s Disease Word List; AFT, Animal Fluency Test; DSST, Digit Symbol Substitution Test.

**Figure 2:**
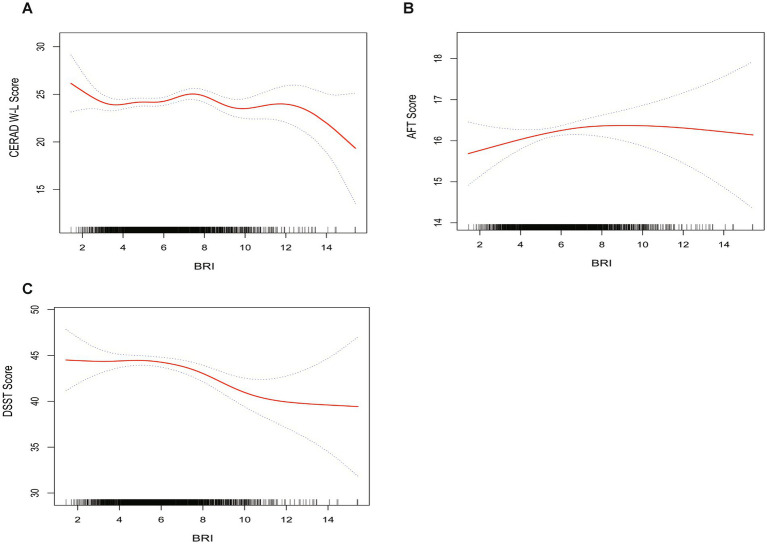
The weighted non-linear relationships between BRI and cognitive function. The solid red line represents the smooth curve fit between variables. Blue bands represent the 95% of confidence interval from the fit. **(A)** BRI and CERAD W-L; **(B)** BRI and AFT; **(C)** BRI and DSST.

From a non-linear perspective, the negative correlation between BRI levels and DSST scores was further corroborated by weighted smooth curve fitting (Figure 2).

### 3.3 Subgroup analysis

Weighted subgroup and interaction analyses were conducted, taking into factors such as sex, race, education level, alcohol intake, smoking status, hypertension, hyperlipidemia, diabetes, stroke history, and depression. The purpose of these studies was to investigate if there are any possible differences between certain subgroups and the general population regarding the relationship between BRI and cognitive performance (Table 3).

**Table 3:** Subgroup analysis of the association between BRI and cognitive function.

Subgroup	CERAD W-L *β* (95%CI), *p*	*p* for interaction	AFT *β*(95%CI), *p*	*p* for interaction	DSST *β*(95%CI), *p*	*p* for interaction
Sex		0.680		0.477		0.040
Male	−0.07 (−0.31, 0.16), 0.533		0.16 (−0.04, 0.35), 0.117		−0.75 (−1.23, −0.27), 0.002	
Female	−0.01 (−0.19, 0.17), 0.890		0.07 (−0.08, 0.22), 0.380		−0.12 (−0.49, 0.25), 0.523	
Race		0.842		0.483		0.993
Mexican American	−0.05 (−0.79, 0.68), 0.885		−0.02 (−0.64, 0.60), 0.946		−0.42 (−1.95, 1.10), 0.586	
Other Races	0.02 (−0.48, 0.52), 0.935		0.39 (−0.02, 0.81), 0.064		−0.33 (−1.36, 0.70), 0.530	
Non-Hispanic White	−0.08 (−0.24, 0.09), 0.355		0.06 (−0.07, 0.20), 0.375		−0.39 (−0.72, −0.06), 0.022	
Non-Hispanic Black	0.15 (−0.35, 0.65), 0.552		0.14 (−0.28, 0.55), 0.522		−0.24 (−1.26, 0.79), 0.652	
Education		0.067		0.796		0.452
<High school	−0.04 (−0.37, 0.30), 0.836		0.14 (−0.14, 0.42), 0.325		0.04 (−0.64, 0.73), 0.901	
High school	0.25 (−0.04, 0.55), 0.096		0.02 (−0.23, 0.27), 0.860		−0.53 (−1.14, 0.08), 0.088	
>High school	−0.16 (−0.34,0.03), 0.097		0.11 (−0.05, 0.26), 0.185		−0.34 (−0.72, 0.04), 0.081	
Alcohol		0.357		0.306		0.224
No	−0.13 (−0.34, 0.09), 0.248		0.16 (−0.02, 0.34), 0.084		−0.14 (−0.58, 0.31), 0.538	
Yes	0.01 (−0.18, 0.20), 0.945		0.03 (−0.12, 0.19), 0.670		−0.50 (−0.90, −0.11), 0.012	
Smoking		0.830		0.249		0.919
No	−0.03 (−0.18, 0.12), 0.740		0.07 (−0.06, 0.19), 0.304		−0.35 (−0.66, −0.04), 0.028	
Yes	−0.08 (−0.53, 0.37), 0.737		0.30 (−0.08, 0.67), 0.121		−0.30 (−1.23, 0.63), 0.529	
Hypertension		0.022		0.322		0.066
No	−0.24 (−0.47, −0.01), 0.041		0.03 (−0.16, 0.23), 0.728		−0.01 (−0.49, 0.47), 0.960	
Yes	0.10 (−0.08, 0.28), 0.291		0.16 (0.11, 0.31), 0.042		−0.58 (−0.96, −0.20), 0.003	
Hyperlipidemia		0.445		0.265		0.440
No	−0.12 (−0.33, 0.10), 0.300		0.02 (−0.16, 0.20), 0.836		−0.47 (−0.93, −0.02), 0.041	
Yes	−0.00 (−0.19, 0.18), 0.963		0.16 (−0.00, 0.31), 0.052		−0.24 (−0.63, 0.15), 0.225	
Diabetes		0.466		0.698		0.734
No	−0.02 (−0.18, 0.14), 0.819		0.09 (−0.04, 0.22), 0.195		−0.37 (−0.69, −0.04), 0.028	
Yes	−0.15 (−0.47, 0.17), 0.357		0.15 (−0.12, 0.41), 0.283		−0.24 (−0.90, 0.42), 0.474	
Stroke		0.136		0.685		0.418
No	−0.02 (−0.17, 0.12), 0.754		0.08 (−0.05, 0.20), 0.214		−0.35 (−0.66, −0.05), 0.024	
Yes	−0.44 (−0.96, 0.09), 0.105		0.17 (−0.27, 0.61), 0.443		−0.81 (−1.90, 0.27), 0.142	
Depression		0.187		0.669		0.296
No	−0.06 (−0.21, 0.09), 0.419		0.09 (−0.03, 0.22), 0.133		−0.28 (−0.59, 0.02), 0.070	
Yes	0.30 (−0.22, 0.81), 0.258		0.19 (−0.24, 0.62), 0.382		−0.87 (−1.94, 0.19), 0.109	

Age, sex, race, education level, PIR, alcohol and smoking use status, hypertension, and hyperlipidemia, diabetes, stroke, and depression were adjusted. PIR, poverty income ratio; CERAD W-L, Consortium to Establish a Registry for Alzheimer’s Disease Word List; AFT, Animal Fluency Test; DSST, Digit Symbol Substitution Test.

According to the weighted subgroup analysis, the relationship between BRI and cognitive performance, particularly in the DSST, showed a significant sex interaction (*p* for interaction = 0.040). In males, the higher BRI was significantly negatively associated with lower DSST scores (*β* = −0.75, 95% CI = −1.23 to −0.27, *p* = 0.002), while no significant association was observed in females (*p* = 0.523), indicating that the negative impact of BRI on cognitive function was more pronounced in males. Additionally, significant negative associations were found in the following weighted subgroups: non-Hispanic White individuals (*β* = −0.39, 95% CI = −0.72 to −0.06, *p* = 0.022), alcohol users (*β* = −0.50, 95% CI = −0.90 to −0.11, *p* = 0.012), non-smokers (*β* = −0.35, 95% CI = −0.66 to −0.04, *p* = 0.028), individuals with hypertension (*β* = −0.58, 95% CI = −0.96 to −0.20, *p* = 0.003), those without hyperlipidemia (*β* = −0.47, 95% CI = −0.93 to −0.02, *p* = 0.041), individuals without diabetes (*β* = −0.37, 95% CI = −0.69 to −0.04, *p* = 0.028), and individuals without a history of stroke (*β* = −0.35, 95% CI = −0.66 to −0.05, *p* = 0.024).

In the weighted analysis, a significant interaction between hypertension status and BRI was observed in the CERAD W-L test (*p* for interaction = 0.022), indicating that individuals without hypertension exhibited a stronger negative association between BRI and cognitive performance compared to those with hypertension. Specifically, individuals without hypertension showed a significant negative association with poorer cognitive performance on the CERAD W-L test (*β* = −0.24, 95% CI = −0.47 to −0.01, *p* = 0.041). In contrast, individuals with hypertension exhibited a significant positive association with cognitive performance on the AFT test (*β* = 0.16, 95% CI = 0.01 to 0.31, *p* = 0.042). These findings suggest that the higher BRI is associated with poorer cognitive performance in certain subgroups, with varying patterns of association across different cognitive tests and specific subgroups.

## 4 Discussion

This cross-sectional study aimed to investigate the correlation between the BRI and cognitive function in a sample of 1,870 older individuals aged 65 and older in the United States. The results reveal a notable negative correlation between elevated BRI and reduced DSST scores, suggesting that increased BRI is associated with greater cognitive decline. This relationship was consistent across various subgroups, particularly among males, non-Hispanic white individuals, alcohol consumers, non-smokers, and those with hypertension, without hyperlipidemia, diabetes, or a history of stroke. Importantly, this is the first study to assess the relationship between BRI and cognitive function in older Americans, suggesting that BRI may serve as a useful indicator for assessing body shape-related cognitive decline in the elderly population.

Numerous studies have shown a significant link between obesity and an increased risk of cognitive impairment (Norris et al., 2023; Peykari, 2021; Ge et al., 2023). These studies support our observation that a higher BRI is significantly correlated with more severe cognitive decline. Large-scale studies in China and the United States have consistently found that overweight or obesity increases the risk of dementia in older adults (Feinkohl et al., 2018; Wang et al., 2019; Espeland et al., 2018). Obesity has been recognized as a major risk factor for AD (Dye et al., 2017; Bischof and Park, 2015). However, variations in sample size and research methodologies across different regions can lead to inconsistent findings. Obesity is closely linked to brain structural changes, including excessive atrophy and white matter lesions (Gustafson et al., 2004; Gustafson et al., 2004; Ward et al., 2005). A prospective study among African Americans found that increased central adiposity was associated with accelerated cognitive decline over a five-year follow-up period (Anand et al., 2022). Additionally, research indicates that cognitive function can improve following weight loss surgery in obese individuals (Vreeken et al., 2023). These findings provide strong evidence supporting the link between obesity and cognitive decline, emphasizing the significance of obesity as a risk factor for cognitive impairment. However, the detrimental impact of obesity on cognitive function is not consistently observed. Recent research on elderly Colombians has found no significant association between overweight or obesity and the progression of cognitive decline (O’Donovan et al., 2022). Some studies suggest that overweight or obesity in old age might confer a protective effect against cognitive impairment or dementia (Qu et al., 2020; Puzianowska-Kuznicka et al., 2019; Hughes et al., 2009; Buchman et al., 2006). These studies primarily used BMI to measure obesity, reflecting overall fat distribution rather than regional fat distribution (Prentice and Jebb, 2001), particularly in older adults (Tchkonia et al., 2010). The limitations of BMI may contribute to these inconsistent findings. BRI, as a novel anthropometric measure, combines weight and WC to provide a more comprehensive assessment of visceral fat. Compared to traditional BMI, BRI better reflects fat distribution and body shape characteristics (Zhang et al., 2024). Recently, Zhang et al. studied the association between ABSI and cognitive function in the elderly U.S. population, finding a significant negative correlation between ABSI and cognitive impairment (Zhang et al., 2024). Compared to ABSI, BRI assesses visceral fat distribution using a simple calculation of WC and height. While ABSI offers a more complex evaluation of central obesity’s relationship with cognitive decline, BRI, as a simpler tool, also demonstrates strong predictive ability. Future studies should compare the applications of ABSI and BRI across diverse populations to determine which index offers greater clinical value in predicting cognitive impairment.

Earlier research has demonstrated a significant link between obesity and cognitive impairment, but the results have been inconsistent, partly due to varying metrics used to measure obesity. BMI may not adequately reflect the specific impact of obesity on cognitive function. In contrast, the BRI, a new metric, provides a more accurate assessment. This study used BRI to confirm a significant negative correlation between obesity and cognitive decline, particularly in the elderly population. Future research should continue to examine the causal relationship between BRI and cognitive function. Employing longitudinal study designs and larger sample sizes will enhance understanding and provide a more reliable basis for early detection and intervention.

Recent studies have revealed the complex biological mechanisms between obesity and cognitive decline. First, the adipose tissue in obese individuals abnormally secretes pro-inflammatory cytokines, including IL-6 and TNF-*α* (Salas-Venegas et al., 2022). Excessive IL-6 induces vascular endothelial growth factor production, promotes nitric oxide synthase release in endothelial cells, disrupting the tight junction proteins of the blood brain barrier (BBB), increasing its permeability (Ali et al., 2022), upregulating BACE1 enzyme expression, and ultimately leading to neuronal apoptosis and memory impairment (Naomi et al., 2023). TNF-α also activates astrocytes and microglia, inhibits excitatory synaptic transmission, impairs hippocampus-dependent learning and memory, and exacerbates damage by inhibiting long-term potentiation (Takahashi et al., 2021). TNF-α inhibits cAMP response element-binding protein activity, disrupts the expression of spatial memory-related genes (including c-FOS, BDNF, and Arc), and weakens neuronal survival and neurogenesis, leading to impaired spatial and contextual fear memory (Naomi et al., 2023). Second, obesity affects cognitive function by regulating leptin and adiponectin levels. Elevated leptin levels trigger leptin resistance, exacerbate insulin resistance by inflammatory responses, and ultimately lead to cognitive impairment (Wong Zhang et al., 2023). Although adiponectin has anti-inflammatory effects, its levels significantly decrease in obesity, increasing the risk of insulin resistance and inflammation, which further aggravates cognitive dysfunction and the risk of AD (Forny-Germano et al., 2018).

Third, obesity not only induces peripheral insulin resistance but may also trigger brain insulin resistance, marked by impaired insulin-induced long-term inhibition and reduced insulin signaling in the brain (Sripetchwandee et al., 2018). Peripheral insulin resistance may promote hepatic lipid production and ceramide formation, contributing to brain insulin resistance (Summers, 2006). Ceramides, composed of sphingosine and fatty acids, can cross the BBB, triggering oxidative stress and inflammation in the brain, ultimately causing neurodegeneration (Tong and de la Monte, 2009). Furthermore, mitochondrial dysfunction in the brain (e.g., excess reactive oxygen species, mitochondrial depolarization, and swelling) is closely related to brain insulin resistance, and these pathological processes may contribute to cognitive decline and increase the risk of AD (Sripetchwandee et al., 2018). Lastly, obesity-induced gut microbiota dysbiosis impairs brain health by promoting systemic inflammation and disrupting the BBB (Wong Zhang et al., 2023). Gut microbiota dysbiosis may alter tryptophan metabolism, affecting serotonin signaling (Whiley et al., 2021). Weakened serotonin signaling may also increase hypothalamic–pituitary–adrenal (HPA) axis activity in dementia patients (Whiley et al., 2021). Increased HPA axis activity raises cortisol levels, potentially reducing hippocampal volume and impairing cognitive function (Wong Zhang et al., 2023). Gut microbiota dysbiosis reduces short-chain fatty acid levels, weakening their anti-inflammatory effects and altering neural signaling, potentially worsening cognitive impairment (Salami, 2021). These biological mechanisms reveal how obesity affects cognitive function through multiple pathways, offering new perspectives for predicting obesity-related cognitive decline and l a basis for future interventions.

Subgroup analysis demonstrated a significant inverse relationship between BRI and DSST scores in males, indicating that the higher BRI adversely affects cognitive function in males. Research shows that obesity affects cognitive function in men and women through distinct mechanisms. Obesity can accelerate cognitive decline in elderly men, partly due to reduces testosterone levels, which have demonstrated neuroprotective effects by reducing amyloid-*β* toxicity and oxidative stress (Noh et al., 2019). A higher BMI may also protect cognitive function in women, possibly due to increased endogenous estrogen levels in adipose tissue. Despite age-related estrogen decline, women may still prevent neuropathology accumulation by maintaining estrogen receptor signaling in the brain (Espeland et al., 2018). These findings suggest that obesity may affect cognitive function in men and women via different biological mechanisms. Racial analysis demonstrated a strong inverse correlation between BRI and DSST scores in non-Hispanic white individuals, but not in other racial groups. This may be attributed to differences in gene expression and metabolic pathways among races, which increase susceptibility to obesity-related cognitive decline. Research indicates that specific variations in the FTO gene are significantly linked with obesity, hypertension, and diabetes in white individuals, but not in black individuals (Yu et al., 2023). Additionally, the ApoEε4 allele increases the risk of AD, whereas the ApoEε2 allele has a protective effect (Dye et al., 2017). Frequency and expression differences of these gene variants among races may explain the varying relationships between BRI and cognitive function.

Furthermore, there was a notable positive link between BRI and DSST scores among those who use alcohol, suggesting that lifestyle factors may influence the relationship between BRI and cognitive function. Studies indicate that chronic alcohol consumption leads to iron accumulation in specific brain regions, triggering microglial activation and the release of pro-inflammatory cytokines and reactive oxygen species (Wilcockson and Roy, 2024). This process damages neurons and surrounding brain tissue, exacerbating cognitive dysfunction (Wilcockson and Roy, 2024). Furthermore, the impact of BRI on cognitive function in hypertensive individuals varies significantly across different cognitive tests. In the CERAD W-L test among non-hypertensive individuals, BRI shows a significant negative association with cognitive function. This could be due to the lack of compensatory mechanisms in non-hypertensive individuals, where the higher BRI may directly contribute to vascular and metabolic dysfunction, impairing the brain’s learning and memory abilities. In the AFT test, BRI in hypertensive individuals is positively associated with cognitive function, possibly due to hypertensive patients’ ability to maintain vascular function through blood flow redistribution (Talman et al., 1994). This compensatory mechanism may help some hypertensive patients preserve cognitive function, mitigating the adverse impact of BRI. In contrast, in the DSST test, BRI in hypertensive individuals is significantly negatively associated with executive function and processing speed. This could be attributed to long-term hypertensive microvascular damage affecting brain white matter, especially in regions linked to executive function (Uiterwijk et al., 2016). Although no significant negative associations between BRI and cognitive function were found in smokers, dyslipidemia, diabetes, stroke, or depression, this may be due to sample size limitations or other confounding factors. Further research is needed to better understand the long-term effects of these chronic conditions on cognitive function. Additionally, it is essential to acknowledge that the data on smoking, alcohol consumption, and comorbidities were self-reported, which may have introduced reporting bias. Self-reported data are susceptible to memory biases, social desirability effects, and differences in interpretation, potentially affecting the reliability of the data. Specifically, for lifestyle factors like smoking and alcohol consumption, participants may underestimate or overestimate their behaviors. In addition, certain comorbidities, such as hypertension and diabetes, may introduce bias due to undiagnosed conditions. Therefore, future studies should incorporate objective clinical measurement to reduce biases arising from self-reported data.

In this study, we found that the association between BRI and cognitive decline in older adults remained significant even after adjusting for depression as a covariate. This suggests that while psychological factors like depression may influence cognitive function, BRI still independently predicts cognitive decline after controlling for confounders. However, unmeasured confounders, including physical activity, dietary habits, genetic factors, and psychosocial variables, may also influence the relationship between BRI and cognitive function. Moreover, mental health conditions, such as anxiety and depression, may heighten the risk of cognitive decline by impairing brain health (Zhang et al., 2024; Lotfi et al., 2022). Therefore, future research should explore how these psychological factors mediate the relationship between BRI and cognitive function. Although BRI holds promise as a non-invasive marker for predicting cognitive decline, its small effect size may limit its use as a standalone predictor. We recommend incorporating BRI into a multifactorial assessment, alongside other key risk factors such as lifestyle, genetic background, and mental health status, for a more comprehensive risk evaluation. Future longitudinal studies and larger sample multivariate analyses are needed to validate the role of BRI and enhance understanding of its applicability in diverse populations.

This study possesses several strengths, notably the use of a sample that use of a nationally representative from the NHANES, ensuring the broad applicability of the findings. Furthermore, it is the first to systematically examine the association between the BRI and cognitive function, underscoring BRI’s potential in predicting cognitive decline. This offers a unique viewpoint and instrument for identifying and addressing cognitive decline in older individuals at an early stage. The study also performed subgroup analyses, corroborating the robustness of the findings across various populations. However, this study has some limitations. First, the cross-sectional study design limits the ability to definitively establish a causal relationship between BRI and cognitive decline. Therefore, future studies should use longitudinal data or experimental designs to further validate these associations. Second, relying on specific cognitive tests may introduce bias, as these tests mainly assess memory and processing speed, potentially overlooking other cognitive domains, which may limit the ability to fully reflect overall cognitive capacity. Future studies should employ more comprehensive cognitive assessment tools to reduce this bias. Additionally, comparing BRI with traditional obesity measures (such as BMI) and exploring their respective strengths and weaknesses will provide a more accurate assessment of BRI’s effectiveness in predicting cognitive decline. Despite rigorous adjustments, fully eliminating the influence of unmeasured confounding factors remains challenging. Future studies should consider using larger sample sizes or multicenter studies to further validate these findings and provide a more comprehensive evaluation of BRI’s applicability in the elderly population.

Despite its limitations, BRI continues to show promise in predicting cognitive decline among the elderly. Integrating BRI into clinical practice could aid in early identification of high-risk individuals for cognitive decline, particularly when obesity is strongly linked to cognitive impairment. Regular BRI monitoring enables the development of personalized interventions, including dietary adjustments, exercise routines, and weight management strategies, tailored to an individual’s obesity status and cognitive risk. In public health interventions, BRI can function as a non-invasive screening tool for monitoring the health of large populations and contribute to the formulation of strategies aimed at preventing cognitive decline. Future studies should further investigate the application of BRI across various clinical settings and populations, while validating its sensitivity and specificity in predicting and monitoring cognitive decline.

## 5 Conclusion

In this cross-sectional study of adults aged 65 and older, the higher BRI was significantly associated with cognitive decline. This study is the first to establish this association in older U.S. adults, suggesting that BRI could be a valuable non-invasive marker for identifying individuals at risk of cognitive decline.

## Data Availability

The datasets presented in this study can be found in online repositories. The names of the repository/repositories and accession number(s) can be found at: www.cdc.gov/nchs/nhanes/.

## Footnotes

^1^ https://www.cdc.gov/nchs/index.htm
